# Foreign Correspondence

**Published:** 1843-12-23

**Authors:** 


### FOREIGN CORRESPONDENCE.

*The School of Medicine—Dr. Royer-Collard and his Address—Scene between the Orator and Dr. Piorry—M. Guérin and his Procés—Protest of the Parisian and Provincial Physicians against his Conduct—Hydropathy and M. Baudens—Dr. Martin-Solon's Treatment of Rheumatism with large doses of Nitrate of Potash—Dr. Trousseau on Paracentesis Thoracis in Acute Pleurisy—Dr. Ricord's Accident—Syphilitic Inoculation—Dr. Segalas—Carbonate of Lime Calculi—Sea Sickness due to Fear—Effects of Tobacco on Consumption—Appointment of M. Malgaigne—Successful Treatment of Puerperal Convulsions by Hemospasy—Gerdy's Experiments on Reduction of Luxation of the Shoulder.*

To the Editor of the Medical Examiner.

Paris, 8*th November*, 1843.

Sir,—On Friday last (Nov. 3d) the School of Medicine opened, with an inaugural address by Dr. Royer-

Collard, the Professor of Legal Medicine. His subject was the necessity of the physical and chemical sciences in medicine. The style was chaste, correct, and sometimes even *spirituel*, and the manner lively and pleasing, but there was nothing new or striking. In the course of the address several caustic remarks were made by the orator on his confrère, Dr. Piorry, and his bizarre nomenclature; and the orator of last year, Dr. Trousseau, was also handled rather severely. On the retirement of the Faculty to their apartment, a scene is reported to have occurred between the orator and the injured professor, which was adjusted after some difficulty, it being understood that the Professor of Internal Pathology should be the next annual orator, and in that way revenge himself. The unity of instruction in the School was much insisted on by Dr. Royer-Collard. Facts are too stubborn for even the eloquence of the Professor to overcome.

M. Guérin's suits have not yet come up at the Salle des Pas-Perdus. The severe laws against the Press, for political purposes, may, it is feared, turn the issue most unjustly in the present case. Guérin has lost all standing with the profession, which is unanimous against him, as you will see by the journals. The provincial physicians are also daily sending in their signatures to the protest of the medical body here against his conduct.

Baudens, Surgeon to the Duke de Nemours, and chief professor at the Military Hospital of Val de Grace, is treating his rheumatic patients with hydropathy, and, he says, successfully. He has published a case of chronic arthritis with ankylosis of most of the principal joints and endocarditis, speedily and entirely cured by this method.

Dr. Martin-Solon read an elaborate memoir at the Academy of Medicine the other day, on the Treatment of Rheumatism by large doses of Nitrate of Potash. According to him, it is easily tolerated by rheumatic patients in doses of from twenty to sixty grammes. It is more successful in mono-articular rheumatism than in poly-articular—often arresting the disease at once, and generally curing it in from three to seven days. If no benefit is derived from it within three days it should be discontinued, and another plan of treatment substituted.

Professor Trousseau read recently a memoir on Paracentesis Thoracis in acute pleurisy, where the abundant effusion threatens the life of the patient. He first attempted to demonstrate the harmlessness of wounds of the chest, where the great vessels are not involved, and to prove also that the introduction of air into the cavity of the healthy pleura was unattended with great danger; and that even when inflammation of the serous membrane existed, if the quantity of air is not great, and the introduction frequent, that but little danger was to be anticipated. He combated the opinion of Dr. Louis, that simple pleurisy is always easily cured, cited several cases in support of its gravity, stating that he believed death to be due in such cases rather to the effusion than immediately to the inflammation. He mentioned the case of a young girl, in whom, in September last, he performed on the ninth day, when the symptoms of suffocation became imminent, paracentesis, with the instrument ordinarily employed in hydrocele. More than a quart of fluid was withdrawn, the wound closed, and the patient recovered. As to the mode of operation, he declared himself a partisan of that by repeated evacuations.

Dr. Ricord, in dressing a chancre some days since, wounded the thumb of his left hand with the scissors. Phlegmonous inflammation followed, and the wound assumed a bad aspect. In order to satisfy his doubts as to the real nature of the wound, he inoculated his left fore-arm with some of the pus. At the end of twenty-four hours a characteristic pustule appeared, which he cauterised with the Vienna Paste. No doubt of the existence of the local infection remained. The original wound became indurated; the cauterised chancre did not. He submitted himself to an antisyphilitic treatment. In the verbal communication which he made to the Academy on the subject, he stated that he wished to practise on himself inoculation, to show, first, how sincere were his convictions on this point, and secondly, to prove that the assertion that he practised inoculation on others from mere curiosity was false, and that he believed it to be a pure question of science and art, resolved in the most satisfactory manner.

Dr. Segalas stated that he had never seen calculi of carbonate of lime, unless the patients had previously taken carbonate of soda. Dr. Leroy d'Etiolles considered the law, as given by Dr. S., as too absolute; he believed that the occurrence of carbonate of lime in calculi was rare, where the alkaline carbonates had not been used. Dr. Caventou inquired of Dr. Segalas whether he had ever seen calculi of the carbonate of lime which did not contain phosphate of lime. Dr. S. answered, that in the few cases that he had met with of calculi of the carbonate of lime the phosphate of the same base was present.

Dr. Guerprat, a French naval surgeon at Brest, believes that sea-sickness is always due to fear. The commission appointed to inquire into his memoir, combated and denied this novel doctrine.

In a recent report on the health of the workmen in the tobacco manufactories in France, by M. Simeon, the director-general of the administration, it is stated that phthisis is exceedingly rare, comparatively, among them. This fact is attested by the physicians of the manufactories at Bordeaux, Havre, Lille, Morlaix and Strasbourg.

M. Malgaigne has been appointed surgeon to the hospital of St. Antoine, situated in the Faubourg of that name.

Dr. Cazeaux has published two cases of puerperal convulsions successfully treated by Hemospasy,* (*les ventouses Junot.*) They were applied upon the leg and thigh several times in the course of the twenty-four hours.

M. Gerdy has lately made some interesting experiments to determine the lesions which may arise from undue efforts in reducing luxations of the shoulder-joint. In May last a patient entered the surgical wards of La Charité with dislocation of the head of the humerus downwards and forwards, of three weeks duration. Three attempts at reduction were made on the morning of the 5th, ineffectually. On the 7th two other efforts were made; the first failed, and during the second, M. Gerdy remarked on the inner face of the arm a cord firmly stretched, which he supposed to be the median

* See Medical Examiner, No. 20, p. 240.

nerve. The patient complained very much, and M. G., fearing the rupture of the nerve, intermitted any further exertions for the time. The following day he made several experiments on the dead body, with the view of ascertaining, 1st, the influence of violent efforts of extension on the various tissues of the limb, as the nerves, muscles, vessels and ligaments; 2d, the influence of extension, where the fore-arm is extended, and where it is flexed. The result was, that forcible extension is capable of rupturing the muscles, but that they do not become tense as soon as the nerves, where the forearm is extended at a right angle with the trunk; that the nerves thus stretched are, first, the median, then the internal cutaneous, then the cubital and radial; that the brachial vessels are less stretched than the nerves; that if the extension be carried too far, the median and internal cutaneous are ruptured. The facility with which the tense nerve or nerves may be recognised beneath the skin, readily announces the danger. These experiments also proved, that when the fore-arm is flexed on the extended arm the muscles become equally involved with the nerves; that they are stretched together, resist together, and are ruptured together.

